# Treatment of Tumors with Vitamin E Suppresses Myeloid Derived Suppressor Cells and Enhances CD8+ T Cell-Mediated Antitumor Effects

**DOI:** 10.1371/journal.pone.0103562

**Published:** 2014-07-29

**Authors:** Tae Heung Kang, Jayne Knoff, Wei-Hsi Yeh, Benjamin Yang, Chenguang Wang, Young Seob Kim, Tae Woo Kim, Tzyy-Choou Wu, Chien-Fu Hung

**Affiliations:** 1 Seoul Department of Immunology, College of Medicine, Konkuk University, Chungju, South Korea; 2 Department of Pathology, Johns Hopkins Medical Institutions, Baltimore, Maryland, United States of America; 3 Department of Obstetrics and Gynecology, Johns Hopkins Medical Institutions, Baltimore, Maryland, United States of America; 4 Department of Molecular Microbiology and Immunology, Johns Hopkins Medical Institutions, Baltimore, Maryland, United States of America; 5 Department of Oncology, Johns Hopkins Medical Institutions, Baltimore, Maryland, United States of America; 6 Department of Biostatistics and Bioinformatics, Sidney Kimmel Cancer Comprehensive Cancer Center, Johns Hopkins University, Baltimore, Maryland, United States of America; 7 Division of Infection and Immunology, Graduate School of Medicine, Korea University, Seoul, South Korea; Ohio State University, United States of America

## Abstract

Vitamin E has been shown to have strong anticarcinogenic properties, including antioxidant characteristics, making it an ideal candidate for use in combination with immunotherapies that modify the tumor microenvironment. The tumor microenvironment contains immunosuppressive components, which can be diminished, and immunogenic components, which can be augmented by immunotherapies in order to generate a productive immune response. In the current study, we employ the α-tocopherol succinate isomer of vitamin E to reduce immunosuppression by myeloid derived suppressor cells (MDSCs) as well as adoptive transfer of antigen-specific CD8+ T cells to generate potent antitumor effects against the HPV16 E7-expressing TC-1 tumor model. We show that vitamin E alone induces necrosis of TC-1 cells and elicits antitumor effects in TC-1 tumor-bearing mice. We further demonstrate that vitamin E reverses the suppression of T cell activation by MDSCs and that this effect is mediated in part by a nitric oxide-dependent mechanism. Additionally, treatment with vitamin E reduces the percentage of MDSCs in tumor loci, and induces a higher percentage of T cells, following T cell adoptive transfer. Finally, we demonstrate that treatment with vitamin E followed by E7-specific T cell adoptive transfer experience elicits potent antitumor effects in tumor-bearing mice. Our data provide additional evidence that vitamin E has anticancer properties and that it has promise for use as an adjuvant in combination with a variety of cancer therapies.

## Introduction

Vitamin E exists as eight distinct isomers, all of which have strong anticarcinogenic properties, including antioxidant and apoptotic characteristics (for review see [1]). Additionally, many epidemiologic studies support the use of vitamin E as a chemopreventive agent [2]–[4]. The isomer α-tocopherol succinate has been recognized as an effective form of vitamin E for use as an adjuvant in cancer therapy for its ability to inhibit proliferation and induce apoptosis in cancer cells (for review see [5]). These properties of vitamin E may make it an ideal supplement to standard cancer treatments such as chemotherapy as well as immunotherapies that modify the tumor microenvironment.

The tumor microenvironment consists of a variety of immunosuppressive and immunogenic components, including immune cells, tumor cells and stromal cells, which act in opposition to each other. Among the immunosuppressive components, are CD11b+ Gr-1+ myeloid derived suppressor cells (MDSCs), which mediate tumor immunosuppression primarily through inducible nitric oxide synthase (iNOS) and arginase 1 (ARG1), leading to T cell apoptosis and depleting nutrients essential for T cell functioning, respectively [6], [7]. Ultimately these MDSC activities result in limited T cell immune responses and infiltration in the tumor loci [8]. Considering the potent immunosuppressive activities of MDSCs, they serve as an ideal target for anticancer immunotherapies. So far, no study has been reported regarding the impact of vitamin E on MDSCs in the tumor microenvironment.

It is well known that CD8+ T cell-mediated immunity is a highly important component of antitumor immune responses. One method to facilitate tumor eradication is to adoptively transfer tumor antigen-specific T cells that have been expanded *ex vivo* (for review see [9]). While naturally occurring tumor infiltrating lymphocytes have been shown to produce clinical response rates in melanoma, in general, other cancers require genetically engineered T cells [10]. Indeed, studies have emerged employing T cells engineered to express an antigen receptor specific for the target antigen with high affinity and/or high specificity. For example, human T cells have been engineered to express mouse T cell receptors (TCRs) and used to target melanoma antigens [11]. Another strategy to generate potent T cells is the use of chimeric antigen receptors (CARs). CARs consist of an antibody variable region gene encoding single chain structures fused to the intracellular domains of TCRs containing T cell activation capabilities [9]. Adoptive T cell transfer methods serve as promising tumor-specific treatments, but they still have room for improvement. For example, the modification of the tumor microenvironment can potentially be used to further improve adoptive T cell transfer immunotherapy.

In the current study, we report an innovative cancer treatment methodology of vitamin E injections combined with antigen-specific adoptive T cell transfer in tumor-bearing mice. We began by characterizing the antitumor effects of vitamin E against HPV 16 E7-expressing TC-1 tumor cells. We found that vitamin E induces TC-1 cell necrosis *in vivo* and reduces tumor volume in TC-1 tumor-bearing mice. Furthermore, we observed CD11b+ Gr-1+ MDSCs accumulated in TC-1 tumor bearing mice mediating suppression of T cell activation and that vitamin E could reverse the T cell suppression. We further examined the mechanism by which vitamin E alleviated the suppressive effects of the MDSCs and found that it was mediated in part by antioxidant activities against nitric oxide. Investigating the effect of vitamin against MDSCs in vivo, we found that vitamin E decreased the percentage of CD11b+ Gr-1+ cells in the tumor loci compared to control DMSO treatment. Finally, we characterized the antitumor effects of vitamin E in combination with adoptive transfer of E7-specific CD8+ T cells. We found that treatment with vitamin E increased the number of E7-specififc T cells in tumor loci. Furthermore, treatment with vitamin E in combination with T cell adoptive transfer induced potent antitumor effects in TC-1 tumor-bearing mice. These results have positive implications for clinical translation.

## Materials and Methods

### Mice

Six- to eight-week-old female C57BL/6 mice were purchased from the National Cancer Institute (Frederick, MD). All animal procedures were performed according to approved protocols by the Johns Hopkins Institutional Animal Care and Committee and in accordance with recommendations for the proper use and care of laboratory animals.

### Cells and reagents

TC-1 cells, an E7-expressing murine tumor model, were generated by co-transformation of primary C57BL/6 mouse lung epithelial cells with HPV-16 E6 and E7 and an activated ras oncogene as previously described [12]. E7 (aa49–57)-specific T cell line [13] and luciferase expressing E7 (aa49–57)-specific T cell line [14], [15] have also been previously described. These cell lines were cultured *in vitro* in PRMI10 (RPMI 1640 supplemented with 10% fetal bovine serum, 50 units/ml of penicillin/streptomycin, 2 mM L-glutamine, 1 mM sodium pyruvate, and 2 mM non-essential amino acids) and grown at 37°C with 5% CO_2_. Vitamin E (D-α-tocopherol succinate) was purchased from Sigma-Aldrich (St Louis, MO).

### *In vitro* and *in vivo* cytotoxicity experiments

For *in vitro* cytotoxicity experiments, 1×10^5^ TC-1 cells per well were added to 24-well plates. Eighteen hours later, tumor cells were treated with Vitamin E (0, 25, 50 uM). After 18 hours, apoptotic (Annexin V^+^ and 7AAD^−^) and necrotic (Annexin V^+^ and 7AAD^+^) cells were measured using PE Annexin V Apoptosis Detection Kit I (BD Pharmingen, San Diego, CA) according to the vendor's protocol. For in vivo cytotoxicity experiments, 1×10^5^ TC-1 cells were injected into C57BL/6 mice (5 per group) subcutaneously. After 10 days, tumor-bearing mice were intraperitoneally injected with vitamin E (2 mg/kg) [16] or DMSO (dimethyl sulfoxide) control. Mice were injected two additional times using the same regimen at 2 day intervals. Tumor growth was monitored by palpation and visual inspection twice a week.

### Myeloid-derived suppressor cells

For the isolation of myeloid-derived suppressor cells (MDSCs), 1×10^5^ TC-1 cells were injected subcutaneously into wild type C57BL/6 mice. 20 days later, splenocytes were collected from naïve (control) or tumor-bearing mice and prepared for flow cytometry analysis to measure CD11b and Gr-1 positive cells. Splenocytes were stained with FITC-conjugated Gr-1 antibody (BD Pharmingen, San Diego, CA) and PE-CD11b antibody. MDSCs were purified by magnetic cell sorting using the mouse CD11b MicroBeads according to the manufacturer's instructions (MACS; Miltenyi Biotec, Auburn, CA). The purity of CD11b^+^ cells obtained by this method was 90–95% and 90% of CD11b^+^ cells were Gr-1^+^ as determined by flow cytometry. For the detection of MDSCs in tumor tissue, TC-1 tumor tissues obtained from tumor-bearing mice were cut into fragments in phosphate-buffered saline (PBS), washed twice with PBS, and then digested with 500 U/ml of Dispase (Godo Shusei, Co., Ltd. Tokyo) at 37°C for 20 min. The supernatants of the digestion were discarded and the remaining fragments were suspended into 5 ml of PBS. The cell suspensions were passed through a stainless wire sieve (100 mesh), washed twice with 20 ml of PBS and centrifuged for 5 min at 15O×g. Pelleted cells were resuspended in PBS and stained with FITC-conjugated Gr-1 antibody (BD Pharmingen, San Diego, CA) and PE-CD11b antibody.

### T cell activation and proliferation

For the T cell activation experiment, 1 µg/ml of CD3 (BD Pharmingen, San Diego, CA) and 0.5 µg/ml of CD28 (BD Pharmingen, San Diego, CA) antibody solutions in PBS were added at a volume of 500 µl/well onto 24 well plate and kept at a temperature of 4°C overnight. One day later, antibodies were washed out and 1×10^5^ CD11b^+^ or CD11b^−^ cells were isolated from the splenocytes of tumor bearing mice and 1×10^6^ splenocytes from naïve mice were incubated with or without 10 µM vitamin E and with 1 µl/ml GolgiPlug (BD Cytofix/Cytoperm Kit) for 16 hours. Cells were then harvested and stained for surface CD8 and intracellular IFN-γ using a previously described standard protocol [17]. For the T cell activation proliferation experiment, 1×10^5^ CD11b^+^ or CD11b^−^ cells from tumor bearing mice and 1×10^6^ splenocytes from naïve mice were prepared as mentioned above. Splenocytes from naïve mice were labeled with 5 µM carboxyfluorescein succinimidyl ester (CFSE) and then incubated with CD11b^+^ or CD11b^−^ cells from tumor bearing mice with or without vitamin E (10 µM) for 3 days. Samples were analyzed on a FACS Calibur flow cytometer, using CellQuest software (Becton Dickinson, San Jose, CA) as described previously [17]. All of the analyses shown were carried out on gated lymphocyte populations.

### Nitric oxide assay

Nitric oxide was measured by QuantiChrom Nitric Oxide Assay Kit (BioAssay Systems) according to the manufacturer's instructions. Briefly, 1×10^5^ CD11b^+^ or CD11b^−^ cells isolated from the splenocytes of tumor-bearing mice were cultured in 96-well plates with or without 25 µM vitamin E. After 18 hours, culture supernatant was collected and nitric oxide was measured according to vendor's protocol.

### Luciferase-based bioluminescence imaging

For the *in vivo* T cell proliferation experiment, mice were injected with 1×10^5^ TC-1 cells and 10 days later, mice were injected with vitamin E (2 mg/kg) or DMSO (control) using intraperitoneal injection for a total of three times at 2 day intervals. Next, 15 days after tumor cell injection, 1×l0^7^ luciferase expressing E7-specific T cells were adoptively transferred intravenously through the tail vein into the mice. One day later, the substrate luciferin was injected intraperitoneally and the level of bioluminescence from cells was detected by IVIS luminescence imaging system series 2000 [14]. Regions of interest from displayed images was designated and quantified as total photon counts using Living Image 2.50 software (Xenogen).

### *In vivo* tumor treatment experiments

For the TC-1 tumor treatment experiment, mice were challenged with 1×l0^5^ TC-1 tumor cells/mouse subcutaneously. 3 days after tumor challenge, mice were injected with vitamin E (2 mg/kg) or DMSO (control) intraperitoneally for a total of three injections at 2 day intervals. 9 days after tumor injection, 1×l0^7^ E7-specific T cells were adoptively transferred intravenously through the tail vein into the mice. Tumor growth was monitored by palpation and visual inspection twice a week.

### Statistical analysis

The data presented in this study are from one representative experiment of the two or three experiments performed, and are expressed as mean ± standard deviation (S.D.). The number of samples in each group for any given experiment was >3. Results for flow cytometry analysis and tumor treatment experiments were evaluated by analysis of variance (one-way ANOVA) and the Tukey-Kramer multiple comparison test. Comparisons between individual data points were performed using Student's t-test.

## Results

### Vitamin E promotes tumor cell necrosis

**Figure 1:**
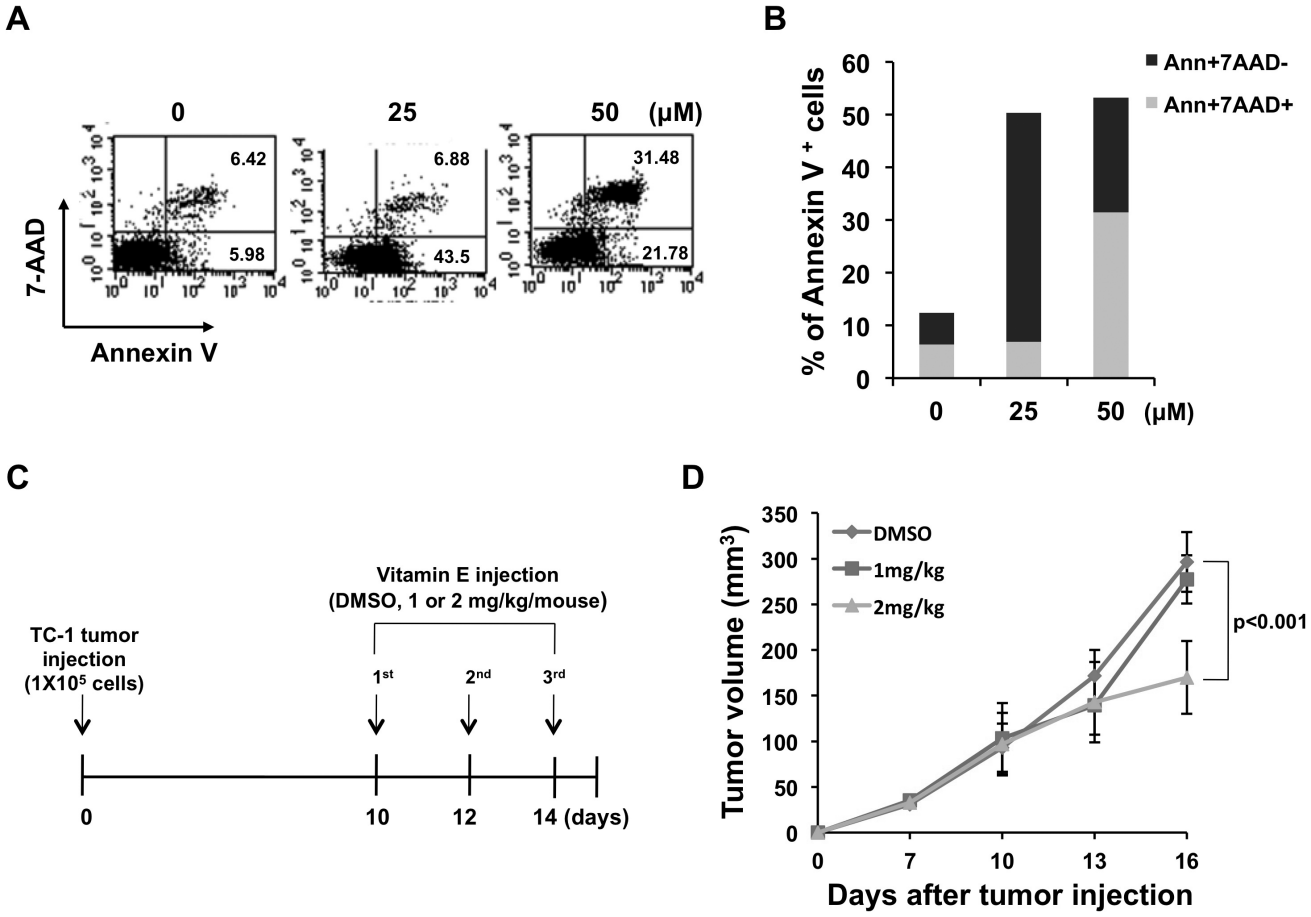
Vitamin E has cytotoxic effect against TC-1 tumor in vitro and in vivo. For the *in vitro* cytotoxicity experiment, 1×10^5^ TC-1 cells were cultured in 24-well plates. Vitamin E (D-α-tocopherol succinate) (0, 25, 50 µM) was administered 18 hours later and apoptotic (Annexin V^+^ and 7AAD^−^) or necrotic (Annexin V^+^ and 7AAD^+^) cells were measured using PE labeled Annexin V and 7AAD. (**A**) Flow cytometry analysis to demonstrate Annexin V^+^ and 7AAD^+^ cells. (**B**) Bar graph depicting the % of Annexin V and 7AAD double positive cells or Annexin V single positive cells. For the *in vivo* cytotoxicity experiment, 1×10^5^ TC-1 cells were injected into C57BL/6 mice subcutaneously. Beginning on day 10, vitamin E (1 or 2 mg/kg) or DMSO (control) was injected intraperitoneally three times at 2 day intervals. (**C**) Schematic diagram of the treatment regimen. (**D**) Line graph depicting growth of TC-1 tumor masses after treatment with vitamin E or DMSO.

We first characterized the antitumor effects toward HPV-16 E7-expressing TC-1 tumor cells generated by vitamin E treatment. TC-1 tumor cells were incubated with 0, 25 or 50 µM vitamin E and subsequently examined for an apoptotic (Annexin V^+^ and 7AAD^−^) or necrotic (Annexin V^+^ and 7AAD^+^) cell marker profile. **Figures 1A** and **B** show that TC-1 cells were increasingly cytotoxic with vitamin E treatment and that treatment with higher vitamin E concentration elicited a higher percentage of necrotic TC-1 cells as opposed to apoptotic cells. To characterize the antitumor effects of vitamin E treatment *in vivo*, mice were subcutaneously challenged with TC-1 tumor cells and then treated with three injections of vitamin E beginning 10 days later as outlined in **Figure 1C**. TC-1 tumor-bearing mice experienced increased antitumor effects, as measured by tumor volume, when treated with 2 mg/kg vitamin E compared to control DMSO treatment (**Figure 1D**). This data indicates that vitamin E is capable of inducing necrosis in TC-1 cells and contributing to tumor control in TC-1 tumor-bearing mice.

### Vitamin E reverses suppression of CD8+ T cells by myeloid derived suppressor cells from tumor-bearing mice in vitro

**Figure 2:**
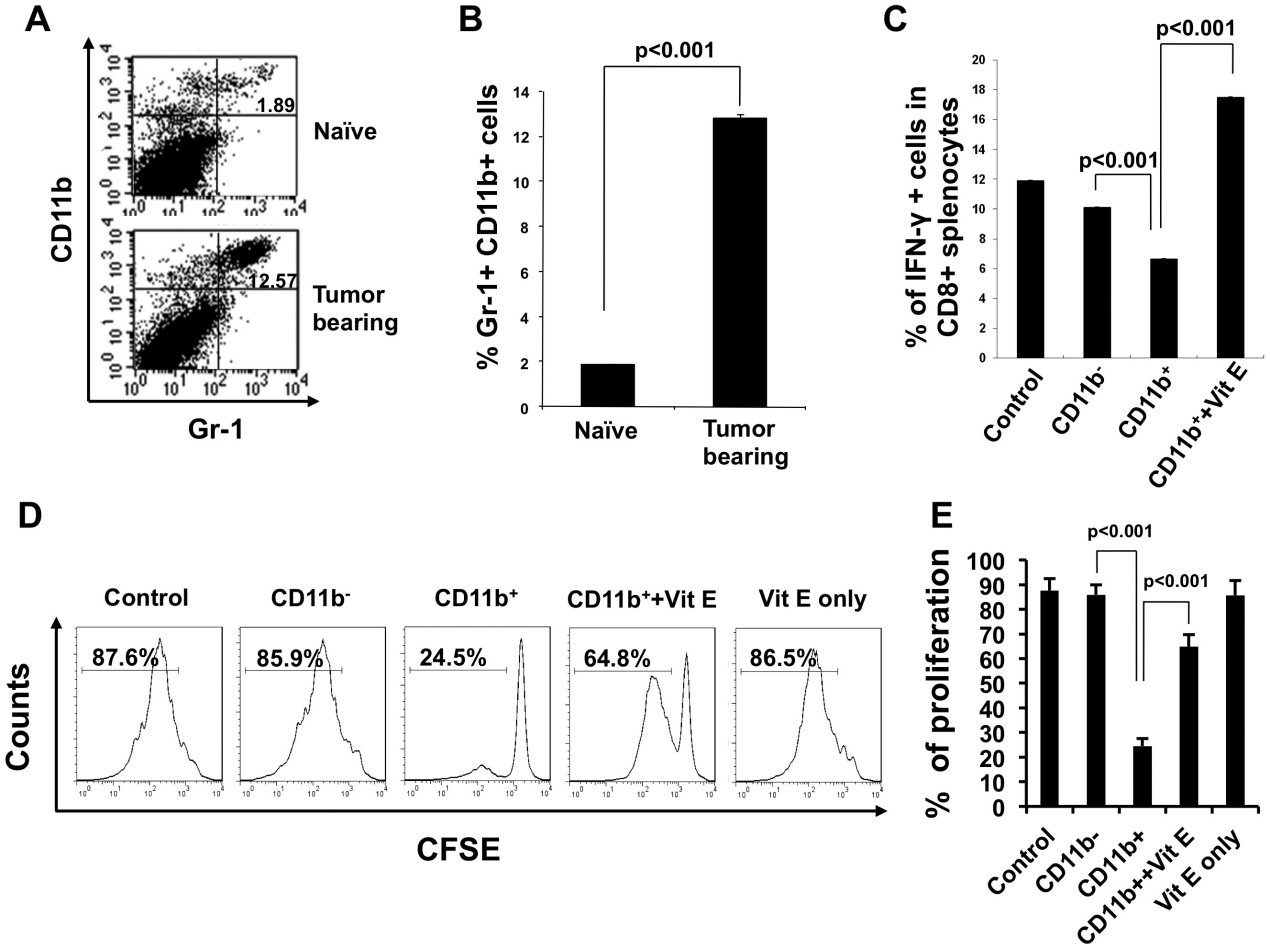
Characterization of myeloid-derived suppressor cells in tumor-bearing mice. 1×10^5^ TC-1 cells were injected subcutaneously into wild type C57BL/6 mice. 20 days later, splenocytes from naïve (control) or tumor-bearing mice were stained with FITC-conjugated Gr-1 antibody and PE-CD11b antibody. (**A**) Flow cytometry analysis to demonstrate CD11b and Gr-1 positive cells. (**B**) Bar graph depicts % of CD11b and Gr-1 positive cells (mean ± SD). Data shown are from one representative experiment of three performed. For the characterization of MDSC-mediated CD8+ T cell suppression, MDSCs were purified by magnetic cell sorting using mouse CD11b MicroBeads. The purity of MDSCs was determined by flow cytometry. Purified MDSCs were used in the T cell activation and proliferation experiment as described in the Materials and Methods. (**C**) Bar graph depicting the % of IFN-γ positive cells in CD8 positive splenocytes (mean ± S.D.). (**D**) Representative flow cytometry histograms showing T-cell proliferation after stimulation with CD3 and CD28 Antibody. (**E**) Bar graph depicting the % of proliferated cells in CD8 and CFSE positive splenocytes (mean ± S.D.).

In order to examine the effect of vitamin E on immunosuppression, we characterized the presence of MDSCs in tumor-bearing mice. Splenocytes from naïve and TC-1 tumor-bearing mice were stained for the MDSC markers CD11b and Gr-1 and analyzed by flow cytometry. **Figure 2A** and **B** show that TC-1 tumor-bearing mice had a significantly higher percentage of CD11b+ Gr-1+ MDSCs among all splenocytes compared to naïve mice. Next, we characterized CD8+ T cell suppression mediated by MDSCs and whether the suppressive effects could be altered by vitamin E. CD8+ T cells were activated using non-specific CD3 and CD28-specific antibodies and were then incubated with CD11b+ or CD11b- cells isolated from tumor-bearing mice with or without vitamin E. As shown in **Figure 2C**, CD8+ T cells incubated with CD11b+ splenocytes were significantly less activated as measured by IFN-γ secretion. Furthermore, the addition of vitamin E to CD8+ T cells incubated with CD11b+ splenocytes reversed CD8+ T cell suppression by MDSCs. In addition, the proliferation of CD8+ T cells incubated with CD11b+ splenocytes after stimulation with CD3 and CD28 antibodies showed a three to four time reduction compared to T cells incubated with CD11b- splenocytes as measured by CFSE dilution. Importantly, vitamin E treatment in CD8+ T cells incubated with CD11b+ splenocytes was able to rescue T cell proliferation (**Figure 2D** and **E**). These data indicate that the antitumor effects generated by vitamin E may be in part due to decreased MDSC-mediated CD8+ T cell suppression.

### Vitamin E has antioxidant effects against nitric oxide

**Figure 3:**
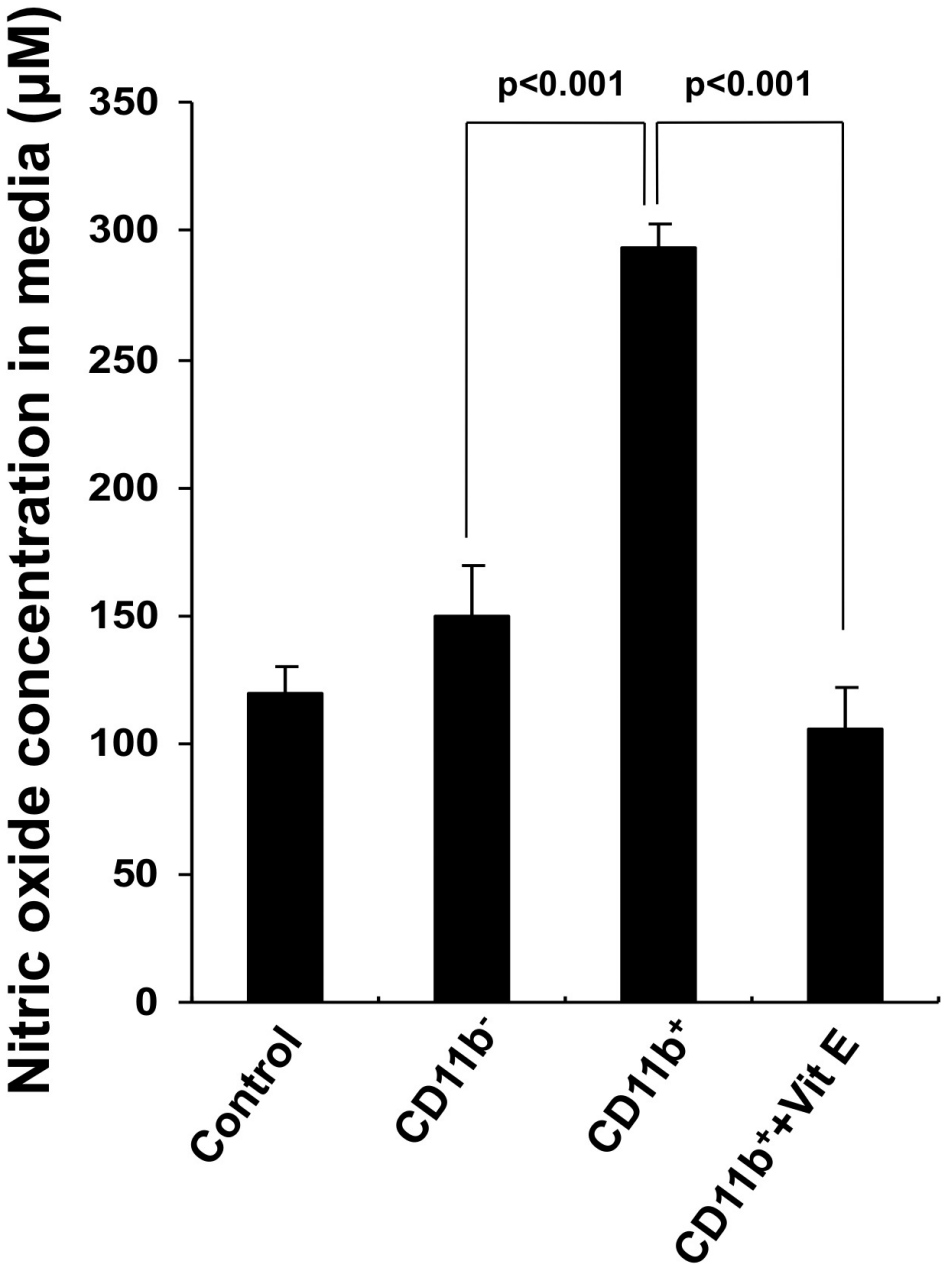
Vitamin E has antioxidant effect against nitric oxide. 1×10^5^ CD11b^+^ or CD11b^−^ cells isolated from splenocytes of tumor-bearing mice were cultured in 96-well plates with or without 25 µM Vitamin E. After 18 hours, culture supernatant was collected and nitric oxide was measured according to vendor's protocol. Bar graph depicts the concentration of nitric oxide in each medium (mean ± S.D.).

MDSC-mediated CD8+ T cell suppression has been shown to be due to a nitric oxide (NO)-dependent mechanism [6]. Therefore, we sought to determine whether NO played a role in what we observed in **Figure 2C**. CD11b+ or CD11b- splenocytes were incubated with or without vitamin E. The concentration of NO in the culture supernatant was measured 18 hours later. **Figure 3** shows that NO concentration was significantly higher in the culture supernatant of CD11b+ cells compared to CD11b- cells, and that the culture supernatant of CD11b+ cells treated with vitamin E had a significantly lower concentration of NO than untreated CD11b+ cells. This data suggests that vitamin E reduces MDSC-mediated CD8+ T cell suppression through a NO-dependent mechanism.

### Vitamin E reduces MDSCs in tumors of TC-1 tumor-bearing mice

**Figure 4:**
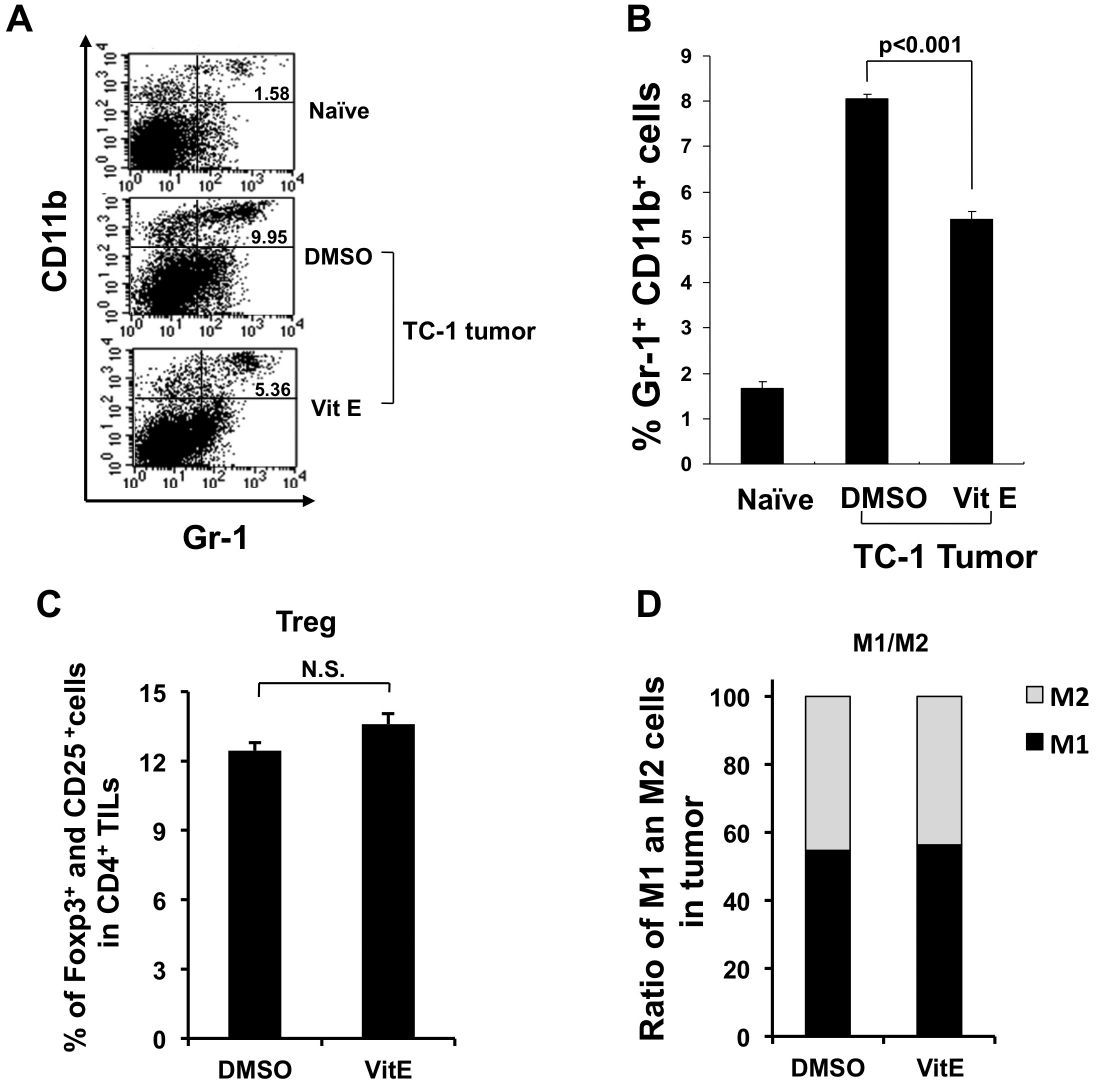
Characterization of myeloid-derived suppressor cells in tumor-bearing mice treated with vitamin E. 1×10^5^ TC-1 cells were injected subcutaneously into wild type C57BL/6 mice. 10 days later, mice were treated using the regimen as described in Figure 1C. Tumor tissue was collected from each group of mice 3 days after the last treatment and prepared for flow cytometry analysis to measure CD11b and Gr-1 positive cells. (**A**) Tumor cells were stained with FITC-Gr-1 antibody and PE-CD11b antibody. (**B**) Bar graph depicts % of CD11b and Gr-1 positive cells (mean ± SD). Data shown are from one representative experiment of three performed. (**C**) Tumor cells were stained with Mouse Regulatory T Cell Staining Kit (eBioscience, San Diego, CA) and bar graph depicts % of Foxp3 and CD25 positive cells in CD4 positive cells. (**D**) Tumor cells were stained with APC-labeled CD11b, FITC labeled F4/80, PE labeled CD206 antibody (eBioscience, San Diego, CA) and bar graph depicts ratio of M1 an M2 cells in CD11b and F4/80 positive cells.

We next examined the effect of vitamin E on MDSCs in the tumor loci of TC-1 tumor-bearing mice. Mice were challenged with TC-1 tumor cells and treated as outlined in **Figure 1C**. Tumor tissue was isolated, stained with CD11b and Gr-1-specific antibodies and analyzed by flow cytometry. As shown in **Figure 4**, TC-1 tumor-bearing mice treated with vitamin E had a significantly lower percentage of CD11b+ Gr-1+ MDSCs in tumor loci compared to control-treated TC-1 tumor-bearing mice. This data demonstrates that vitamin E not only decreases MDSCs systemically, but also in tumor loci. Furthermore, the percentage of CD25+ FoxP3+ CD4+ regulatory T cells in mice treated with vitamin E is comparable to the percentage in mice treated with DMSO (**Figure 4C**). In addition, no difference between the ratio of alternatively activated macrophages (M2 macrophages) over classically activated macrophages (M1 macrophages) in mice treated with vitamin E versus mice treated with DMSO were observed (**Figure 4D**). These results show that vitamin E's reversion of immunosuppressive effects is specific to MDSCs and not to other classes of immune suppressive cells.

### Vitamin E enhances T cell accumulation in TC-1 tumor-bearing mice

**Figure 5:**
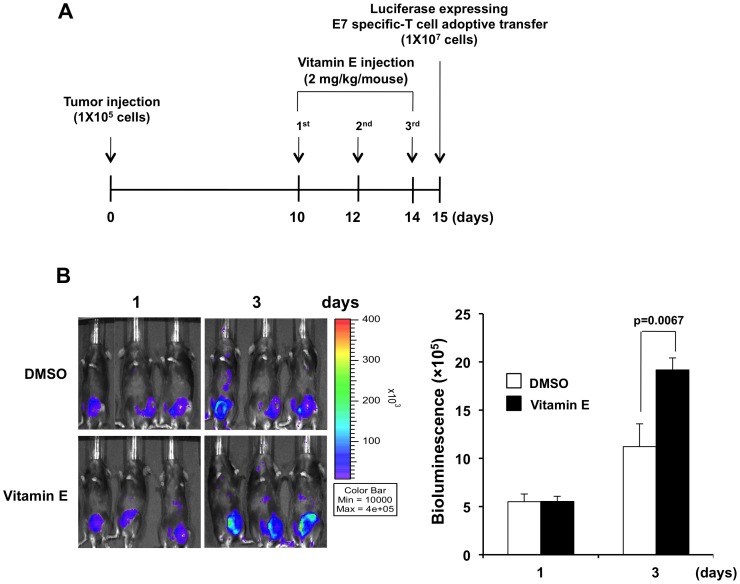
Characterization of the T-cell accumulation in tumor-bearing mice treated with Vitamin E. 1×10^5^ TC-1 cells were injected subcutaneously into wild type C57BL/6 mice. 10 days later, mice were treated as in (A) and one day after the last vitamin E treatment, luciferase-expressing E7-specific CD8+ T-cells were adoptively transferred using intravenous injection. (**A**) Schematic diagram of the treatment regimen. (**B**) Bar graph depicting the fluorescence intensity in tumor-bearing mice treated with vitamin E or DMSO (mean ± S.D.).

We then further characterized the effect of vitamin E on CD8+ T cells by specifically examining T cell accumulation in tumor loci. Tumor-bearing mice were injected with vitamin E three times and subsequently received adoptive transfer of luciferase-expressing E7-specific CD8+ T cells as shown in **Figure 5A**. Mice were monitored for E7-specific CD8+ T cell presence by luminescence imaging. We observed that 3 days after T cell adoptive transfer, TC-1 tumor-bearing mice treated with vitamin E had significantly higher bioluminescence activity indicating a higher number of T cells in tumor loci (**Figure 5B**). It is likely that the increase in CD8+ T cells within the tumor is due to enhanced proliferation enabled by vitamin E-mediated alleviation of suppression.

### Treatment combining vitamin E and T cell adoptive transfer generates synergistic antitumor effects in TC-1 tumor-bearing mice

**Figure 6:**
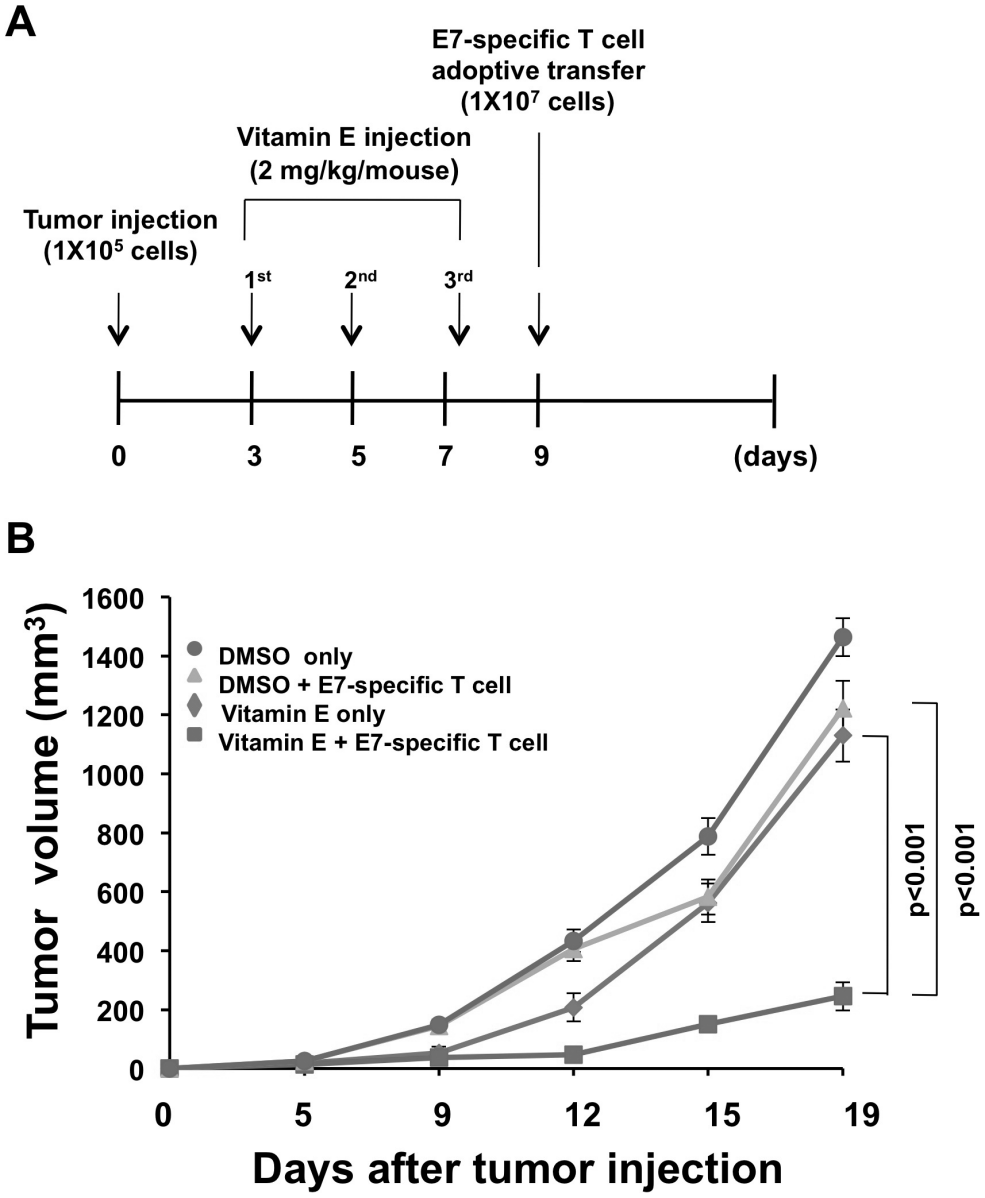
Combined treatment using Vitamin E and T cell adoptive transfer generates synergistic anti-tumor effect in tumor-bearing mice. C57BL/6 mice were injected with 1×10^5^ TC-1 cells/mouse subcutaneously. 3 days later, tumor bearing mice were treated with 2 mg/kg of vitamin E at two day intervals for a total of three times. 2 days after the last treatment, 1×10^7^ E7-specific T cells were adoptively transferred into the tumor-bearing mice. (**A**) Schematic diagram of the treatment regimen. (B) Line graph depicts the growth of TC-1 tumor in different treatment groups over time.

Finally, we examined the efficacy of a combined treatment consisting of vitamin E and T cell adoptive transfer to generate antitumor effects in TC-1 tumor-bearing mice. Mice were challenged with TC-1 tumor cells, given three vitamin E injections and then adoptively transferred with E7-specific CD8+ T cells according to the schedule outlined in **Figure 6A**. **Figure 6B** shows that the combined treatment of vitamin E and subsequent adoptive T cell transfer generated significantly more potent antitumor effects than either vitamin E alone or DMSO combined with adoptive T cell transfer. This data suggests that vitamin E may be a particularly potent therapeutic agent when combined with antigen-specific immune responses.

## Discussion

In the current study, we used the TC-1 tumor model to characterize the antitumor effects of vitamin E in mice. We demonstrated that vitamin E alone induced TC-1 cell necrosis and/or apoptosis in vitro and significantly diminished tumor volume in TC-1 tumor-bearing mice. Furthermore, we showed that vitamin E alleviated the suppression of CD8+ T cell activation mediated by CD11b+ MDSCs, and that this effect was mediated by an NO-dependent mechanism. In addition, we showed that vitamin E treatment decreased the percentage of CD11b+ Gr-1+ MDSCs among splenocytes in TC-1 tumor-bearing mice. We also found that tumor-bearing mice that were treated with vitamin E and received adoptive transfer of T cells generated a significantly greater accumulation of T cells in tumor loci compared to control-treated mice, resulting in potent antitumor effects. Because vitamin E is known to be a potent antioxidant (for review see [18]) and ROS/RNS generated by MDSCs are important for their immunosuppressive function, the observed antitumor effect elicited by vitamin E treatment was likely contributed by its alleviation of ROS/RNS-mediated immunosuppression by MDSCs.

Although vitamin E is most commonly administered orally as a dietary supplement, previous reports on oral treatment of vitamin E did not measure serum tocopherol levels [19], thus making determination of the pharmacokinetics difficult. Studies have also shown that intraperitoneal parenteral administration makes it a lot more plausible to control for the desired dosage [20], [19]. Therefore, we have chosen intraperitoneal injection as our mean of administration in the current study.

In this study, we showed that vitamin E is able to reduce MDSCs mediated suppression of CD8+ T cell activation and proliferation, thereby enhancing cellular immune responses. We also examined vitamin E's effect on humoral immune responses. The result showed that vitamin E is able to generate higher antibody production against antigens in vaccinated mice compared to vaccinated mice receiving only DMSO (**Figure S1**). This suggests that vitamin E treatment is able to induce both cellular and humoral immunity.

Interestingly, the reduction of MDSCs population as a result of vitamin E treatment has lead to an increase in global immunity. There were higher percentages of both CD4+ and CD8+ T cell populations in both the spleen and the tumor in mice treated with vitamin E compared to mice treated with DMSO (**Figure S2**). These results suggest that vitamin E is able to promote higher CD4+ and CD8+ T cells both locally and systemically, which may lead to enhanced tumor protection.

Numerous antioxidants have bee shown to have antitumor effects like vitamin E. For example, olive oil phenols have been shown to inhibit proliferation and promote apoptosis of leukemia, colorectal cancer and breast cancer cell lines (for review see [21]). Furthermore, resveratrol, another dietary phenol, has been recognized as an antioxidant with anticancer properties, including inducing apoptosis of prostate, breast, colon, brain, endometrium, blood, rectum, pancreas, skin, lung, liver, ovary, and bladder cancer cell lines (for review see [22]). Polysaccharides from the *Astragalus membranaceus* plant have been shown to have antitumor and antioxidant effects (for review see [23]).

The encouraging antitumor results from the combination of vitamin E treatment and adoptive immunotherapy consisting of antigen-specific CD8+ T cells suggest that vitamin E treatment may also be used in conjunction with active immunization with antigen-specific tumor vaccines to generate potent antitumor effects. Several therapeutic HPV DNA vaccines have been shown to be capable of generating potent HPV E7-specific CD8+ T cell responses against E7-expressing tumors [24]–[26]. These therapeutic HPV DNA vaccines can potentially be used in conjunction with vitamin E treatment to further enhance therapeutic antitumor effects.

The current study holds substantial promise for clinical translation although additional studies will need to be performed. It will be important to determine the extent of the effects of vitamin E on the tumor microenvironment. Vitamin E may change the chemokine or cytokine profile in the tumor microenvironment. For example, evidence shows that a high dose of dietary vitamin E supplementation in colorectal cancer patients elicits an increase in production of IL2 and IFN-γ by Th1 helper T-cells [27]. Such activities may influence the migration and trafficking of T cells to the tumor. In addition, future studies should also examine the direct effect of vitamin E treatment on proliferation and activation of tumor-specific T cells. Vitamin E may also be cyotoxic to T cells at a very high dose. It will be important to determine the optimal dose of vitamin E to elicit antitumor effects while remaining non-toxic. Conflicting results regarding the chemopreventive effects of vitamin E have been reported. Some reports suggest that, “vitamin E as ingested in diet or in supplements that are rich in γ- or δ-tocopherols is cancer preventive whereas supplementation with high doses of α-tocopherol is not” [28]. Thus, future studies will be needed to determine the optimal dose for use in combination with cancer immunotherapy. In the current study, we found that T cell transfer following vitamin E injections generated potent antitumor effects. It will be important to compare this regimen with one that calls for adoptive T cell transfer prior to vitamin E administration in order to determine optimal regimen for vitamin E treatment.

In conclusion, the current study provides evidence suggesting that vitamin E enhances the antitumor effects of tumor-specific CD8+ T cells by alleviating the suppression of T cell activation by myeloid derived suppressor cells. These results also imply that vitamin E may be combined with other immunotherapeutic strategies employing tumor-specific CD8+ T cell-mediated immune responses. The ability of vitamin E to modify the tumor microenvironment may render it a reagent potentially ideal for use with a variety of cancer therapies.

## Supporting Information

Figure S1 **Vitamin E treatment enhances antibody production.** C57BL/6 mice were treated with 100 µg of ovalbumin protein at 1week intervals three times subcutaneously with 2 mg/kg of vitamin E, without or DMSO. Sera were prepared from mice on day 7 after final immunization. The presence of anti-ovalbumin antibody in the sera was characterized by a direct ELISA as described previously [J Virol, 75 (2001), pp. 2368–2376]. The ELISA plate was read with a standard ELISA reader at 450 nm.
(TIF)

Figure S2 **Vitamin E treatment increases CD4 and CD8 cell population in tumor and spleen.** 1×10^5^ TC-1 cells were injected subcutaneously into wild type C57BL/6 mice. 10 days later, mice were treated using the regimen as described in Figure 1C. Tumor tissue and splenocytes were collected from each group of mice 3 days after the last treatment and prepared for flow cytometry analysis to measure CD4 or CD8 T cells. Splenocytes and tumor cells were stained PE-CD3 and FITC-CD4 or FITC-CD8 antibody. Bar graph depicts % of CD3 and CD4 (**A** and **C**) or CD8 (**B** and **D**) positive cells (mean ± SD). Data shown are from one representative experiment of three performed.
(TIF)
