# Supplementary figures and images for: Treatment of Tumors with Vitamin E Suppresses Myeloid Derived Suppressor Cells and Enhances CD8+ T Cell-Mediated Antitumor Effects
Source: PLoS One. 2014 Jul 29;9(7):e103562. doi: 10.1371/journal.pone.0103562 (PMC4114790; doi:10.1371/journal.pone.0103562)

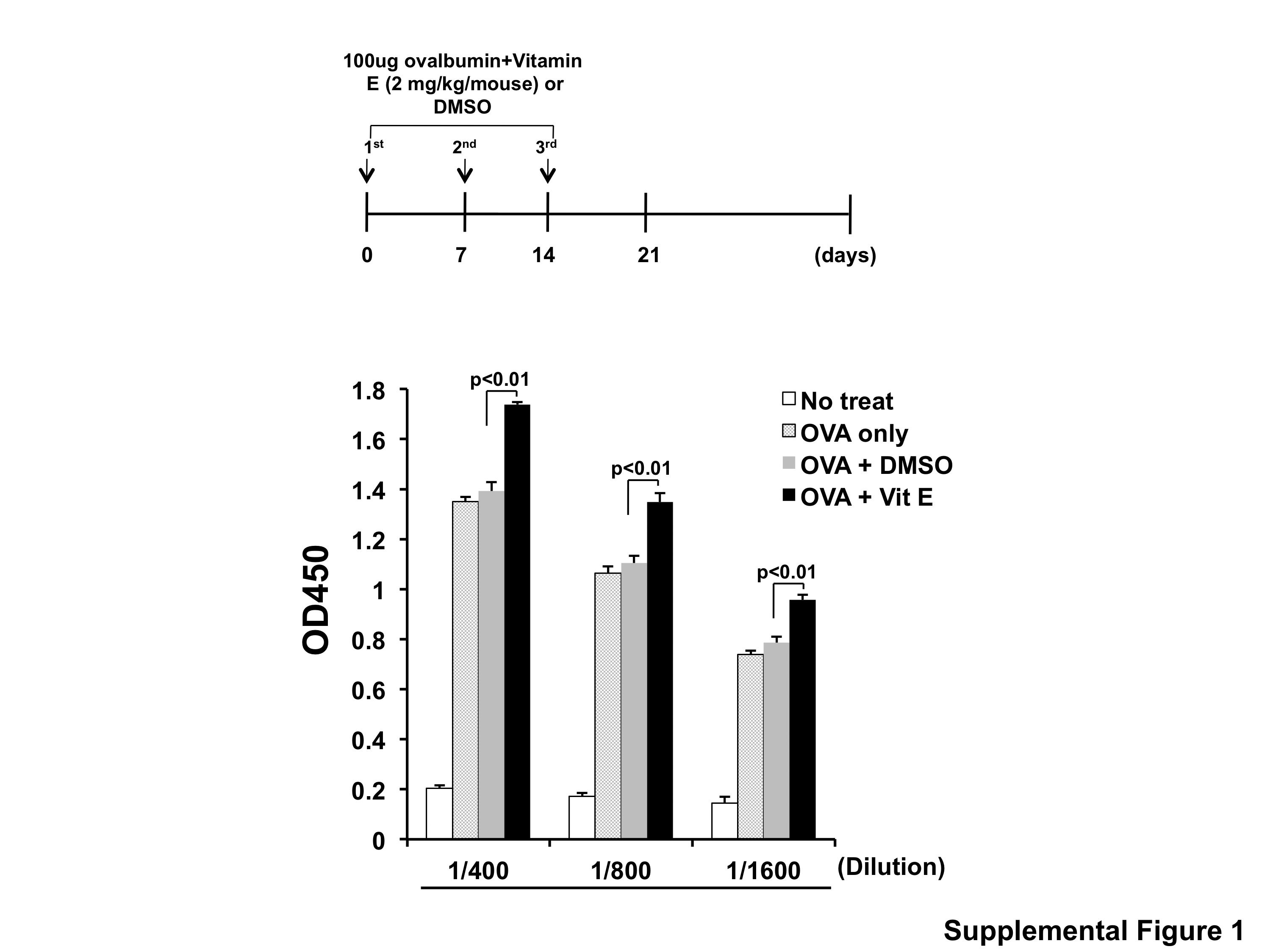

Supplement: Figure S1 — Vitamin E treatment enhances antibody production. C57BL/6 mice were treated with 100 µg of ovalbumin protein at 1week intervals three times subcutaneously with 2 mg/kg of vitamin E, without or DMSO. Sera were prepared from mice on day 7 after final immunization. The presence of anti-ovalbumin antibody in the sera was characterized by a direct ELISA as described previously [J Virol, 75 (2001), pp. 2368–2376]. The ELISA plate was read with a standard ELISA reader at 450 nm. (TIF) [file pone.0103562.s001.tif]

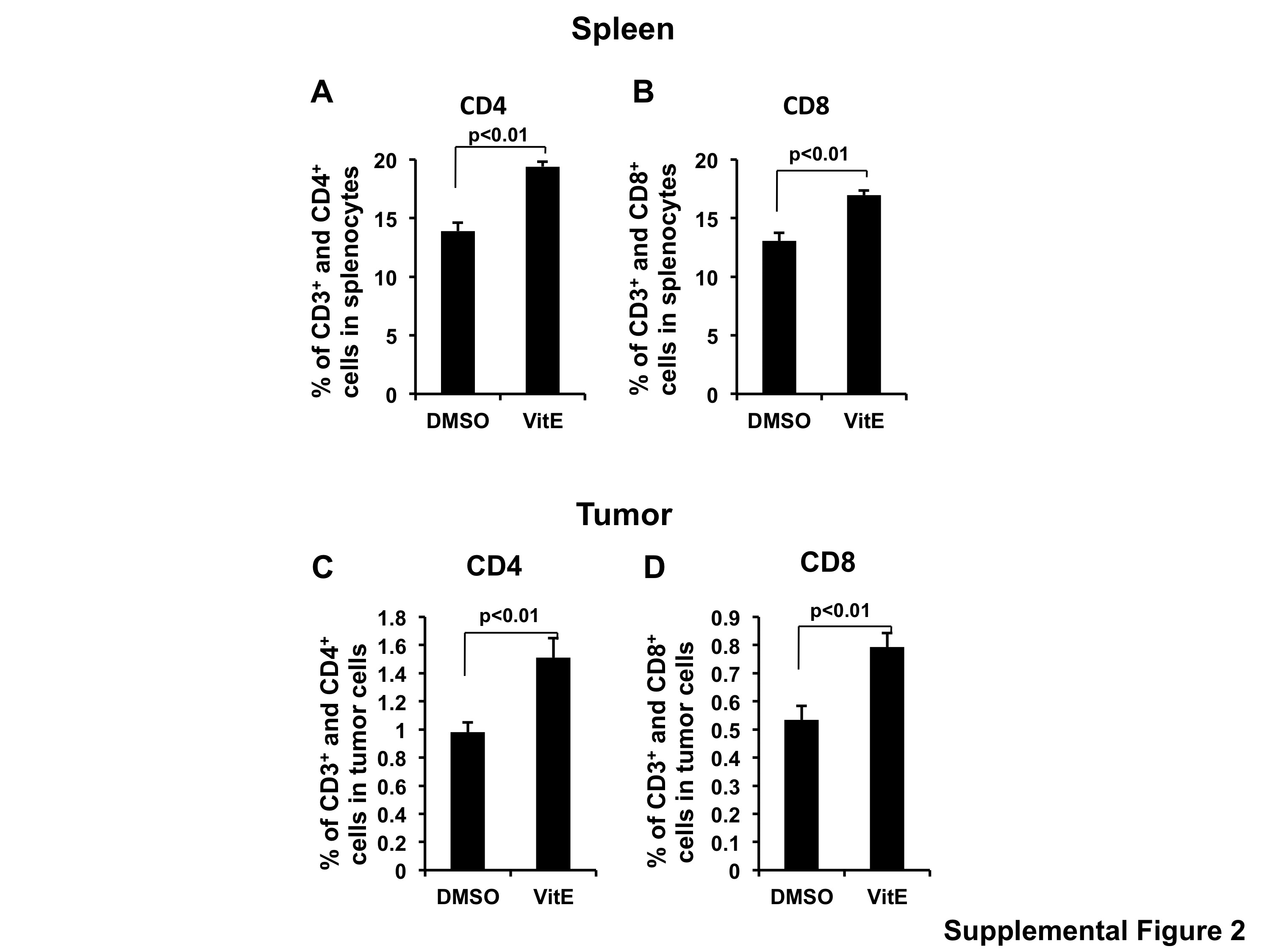

Supplement: Figure S2 — Vitamin E treatment increases CD4 and CD8 cell population in tumor and spleen. 1×10^5^ TC-1 cells were injected subcutaneously into wild type C57BL/6 mice. 10 days later, mice were treated using the regimen as described in Figure 1C. Tumor tissue and splenocytes were collected from each group of mice 3 days after the last treatment and prepared for flow cytometry analysis to measure CD4 or CD8 T cells. Splenocytes and tumor cells were stained PE-CD3 and FITC-CD4 or FITC-CD8 antibody. Bar graph depicts % of CD3 and CD4 (A and C) or CD8 (B and D) positive cells (mean ± SD). Data shown are from one representative experiment of three performed. (TIF) [file pone.0103562.s002.tif]
